# The critical role of BTRC in hepatic steatosis as an ATGL E3 ligase

**DOI:** 10.1093/jmcb/mjad064

**Published:** 2023-10-23

**Authors:** Weiwei Qi, Zhenzhen Fang, Chuanghua Luo, Honghai Hong, Yanlan Long, Zhiyu Dai, Junxi Liu, Yongcheng Zeng, Ti Zhou, Yong Xia, Xia Yang, Guoquan Gao

**Affiliations:** Department of Biochemistry, Zhongshan School of Medicine, Sun Yat-sen University, Guangzhou 510080, China; Department of Biochemistry, Zhongshan School of Medicine, Sun Yat-sen University, Guangzhou 510080, China; Department of Biochemistry, Zhongshan School of Medicine, Sun Yat-sen University, Guangzhou 510080, China; Department of Biochemistry, Zhongshan School of Medicine, Sun Yat-sen University, Guangzhou 510080, China; Department of Clinical Laboratory, The Third Affiliated Hospital of Guangzhou Medical University, Guangzhou 510006, China; Department of Biochemistry, Zhongshan School of Medicine, Sun Yat-sen University, Guangzhou 510080, China; Department of Internal Medicine, University of Arizona College of Medicine, Phoenix, AZ 85004, USA; Department of Biochemistry, Zhongshan School of Medicine, Sun Yat-sen University, Guangzhou 510080, China; Department of Biochemistry, Zhongshan School of Medicine, Sun Yat-sen University, Guangzhou 510080, China; Department of Biochemistry, Zhongshan School of Medicine, Sun Yat-sen University, Guangzhou 510080, China; Department of Clinical Laboratory, The Third Affiliated Hospital of Guangzhou Medical University, Guangzhou 510006, China; Department of Biochemistry, Zhongshan School of Medicine, Sun Yat-sen University, Guangzhou 510080, China; Guangdong Engineering & Technology Research Center for Gene Manipulation and Biomacromolecular Products, Sun Yat-sen University, Guangzhou 510080, China; Department of Biochemistry, Zhongshan School of Medicine, Sun Yat-sen University, Guangzhou 510080, China; Guangdong Province Key Laboratory of Brain Function and Disease, Zhongshan School of Medicine, Sun Yat-sen University, Guangzhou 510080, China; Key Laboratory of Tropical Disease Control, Ministry of Education, Sun Yat-sen University, Guangzhou 510080, China

**Keywords:** adipose triglyceride lipase (ATGL), beta-transducin repeat containing (BTRC), non-alcoholic fatty liver disease (NAFLD), obesity, proteasomal degradation

## Abstract

Non-alcoholic fatty liver disease (NAFLD), characterized by hepatic steatosis, is one of the commonest causes of liver dysfunction. Adipose triglyceride lipase (ATGL) is closely related to lipid turnover and hepatic steatosis as the speed-limited triacylglycerol lipase in liver lipolysis. However, the expression and regulation of ATGL in NAFLD remain unclear. Herein, our results showed that ATGL protein levels were decreased in the liver tissues of high-fat diet (HFD)-fed mice, naturally obese mice, and cholangioma/hepatic carcinoma patients with hepatic steatosis, as well as in the oleic acid-induced hepatic steatosis cell model, while ATGL mRNA levels were not changed. ATGL protein was mainly degraded through the proteasome pathway in hepatocytes. Beta-transducin repeat containing (BTRC) was upregulated and negatively correlated with the decreased ATGL level in these hepatic steatosis models. Consequently, BTRC was identified as the E3 ligase for ATGL through predominant ubiquitination at the lysine 135 residue. Moreover, adenovirus-mediated knockdown of BTRC ameliorated steatosis in HFD-fed mouse livers and oleic acid-treated liver cells via upregulating the ATGL level. Taken together, BTRC plays a crucial role in hepatic steatosis as a new ATGL E3 ligase and may serve as a potential therapeutic target for treating NAFLD.

## Introduction

Non-alcoholic fatty liver disease (NAFLD), characterized by hepatic steatosis and lipid storage, is one of the most frequent liver diseases globally (Diehl and Day, 2017). The disease can present with different clinical phenotypes ranging from hepatic steatosis to non-alcoholic steatohepatitis (NASH), which may progress to liver fibrosis and cirrhosis (Tilg and Targher, 2021). NAFLD increases the risk of type 2 diabetes mellitus, cardiovascular and cardiac diseases, and chronic kidney disease (Byrne and Targher, 2015). Recent studies showed that NAFLD was associated with a 1.93-fold higher relative risk of overall mortality and a high 20-year absolute excess risk (15.3%) primarily due to the increased cancer- and cirrhosis-specific mortality (Simon et al., 2021). Therefore, it is essential to explore the detailed molecular mechanisms underlying the occurrence of NAFLD.

The aberrances in hepatic lipid metabolic pathways, including defective lipolysis, decreased lipid export, excessive free fatty acid (FFA) uptake, and abnormally enhanced *de novo* fatty acid synthesis, may contribute to the development of hepatic steatosis (Fuchs et al., 2014; Foulds et al., 2017). The hydrolysis of triacylglycerol (TAG) to generate FFAs and glycerol is a sequential process involving at least three lipases: adipose triglyceride lipase (ATGL) initiates the lipolysis process, converting TAGs to diacylglycerols, and then hormone-sensitive lipase mediates the hydrolysis of diacylglycerols to monoacylglycerols, which can be further hydrolyzed by monoglyceride lipase (Belfrage et al., 1978). ATGL belongs to the patatin-like phospholipase domain-containing protein family, which includes nine human and eight murine members (Zimmermann et al., 2004; Zechner et al., 2005), and is considered the rate-limiting cytoplasmic TAG lipase in the process of lipolysis (Ong et al., 2011; Sathyanarayan et al., 2017). ShRNA knockdown or genetic ablation of hepatic ATGL promoted progressive hepatic steatosis and enhanced lipid droplet (LD) accumulation (Ong et al., 2011; Wu et al., 2012), while overexpression of ATGL in the liver alleviated steatosis (Turpin et al., 2011). Additionally, NAFLD patients with insulin resistance exhibited higher liver steatosis grades and lower ATGL expression levels (Kato et al., 2008). These studies underscored the significance of lipolysis and ATGL levels in regulating hepatic lipid metabolism. To date, most studies on hepatic ATGL have focused on its enzyme activity and transcriptional regulation (Kralisch et al., 2005; Lass et al., 2006), but little is known about its post-transcriptional regulation and consequent contribution to the development of hepatic steatosis.

The ubiquitin (Ub)-dependent proteasomal degradation pathway plays a pivotal role in cellular protein turnover (Wilson and Cerione, 2000). Ubiquitin-mediated proteolysis impacts various cellular processes such as cell cycle progression, transcription, antigen presentation, receptor endocytosis, fate determination, and signal transduction (Sommer and Seufert, 1992). The stepwise ubiquitination of a target protein involves three kinds of enzymes: E1 Ub-activating enzymes, E2 Ub-conjugating enzymes, and E3 Ub ligases (Cai et al., 2018; Yang et al., 2018). Beta-transducin repeat containing (BTRC), also called β-transducin repeat-containing protein (β-TrCP), is a member of the F-box protein family and serves as a substrate recognition component of E3 Ub ligases (Zheng et al., 2016). It has been shown that BTRC can regulate multiple biological processes by recognizing a wide range of cellular targets for degradation, including cell cycle regulators Wee1 and Cdc25A, the negative regulator of NF-κB signaling IκBα, and other important signal transduction molecules such as β-catenin and Snail (Lau et al., 2012). However, it remains unknown whether BTRC could act as an E3 ligase of ATGL for degradation and play a role in lipid metabolism and hepatic steatosis.

In this study, we demonstrated the role of BTRC as a novel E3 ligase in ATGL degradation and hepatic steatosis. Moreover, knockdown of BTRC could increase ATGL levels, inhibit TAG accumulation, and ameliorate hepatic steatosis, suggesting the potential of targeting BTRC for the treatment of NAFLD.

## Results

### ATGL protein level is downregulated in hepatic steatosis

**Figure 1:**
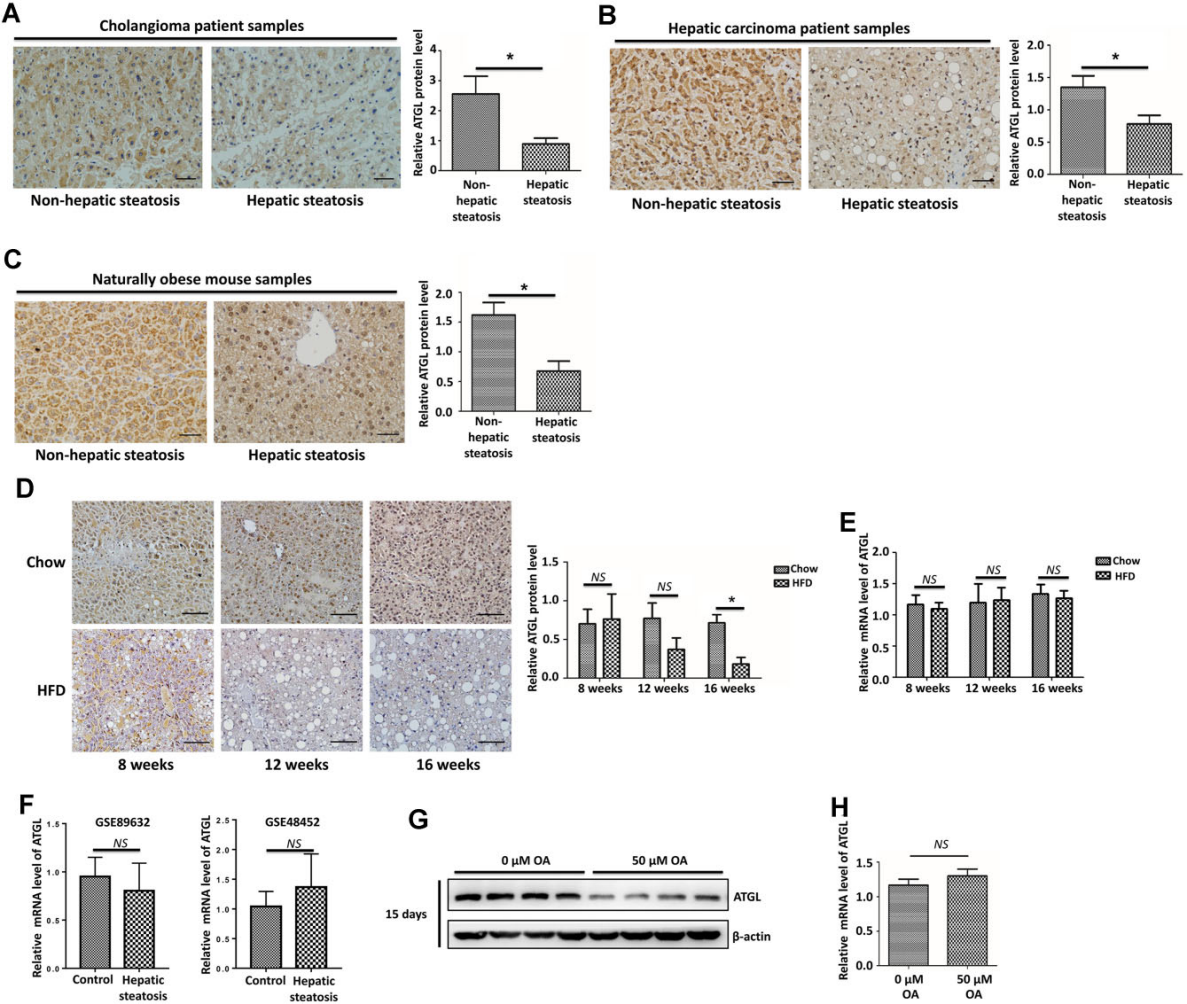
ATGL protein level is downregulated in hepatic steatosis. (**A**–**D**) ATGL protein levels in the liver tissues of cholangioma patients (**A**, *n =* 3 samples/group), hepatic carcinoma patients (**B**, *n =* 11 samples/group), and naturally obese mice (**C**, *n =* 6 mice/group) were determined by IHC. (**D** and **E**) Mice were fed on a chow diet or HFD for 8, 12, and 16 weeks (*n =* 6 mice/group). ATGL protein (**D**) and mRNA (**E**) levels in mouse liver tissues were examined by IHC and qRT-PCR, respectively. (**F**) ATGL mRNA levels in the human liver tissue expression profiling datasets GSE89632 (control, *n =* 23; hepatic steatosis, *n =* 18) and GSE 48452 (control, *n =* 12; hepatic steatosis, *n =* 9). (**G** and **H**) HepG2 cells were treated with 50 μM OA for 15 days. ATGL protein (**G**) and mRNA (**H**) levels were examined by western blotting and qRT-PCR, respectively. β-actin was used as a loading control. Scale bar, 50 μm (**A**–**C**) and 100 μm (**D**). Mean ± SEM; NS, no significance; *P < 0.05.

To assess ATGL protein expression in hepatic steatosis, surgical specimens of patients with hepatic steatosis and non-hepatic steatosis were collected for immunohistochemistry (IHC) analysis. ATGL was significantly downregulated in cholangioma patients with hepatic steatosis (Figure 1A). Similar results were found in paracancerous liver tissues of hepatic carcinoma patients with hepatic steatosis (Figure 1B). Furthermore, we collected 6-month-old naturally obese mice to exclude the effect of the high-fat diet (HFD), and found liver ATGL expression in the obesity group with hepatic steatosis was significantly reduced compared with the control group (Figure 1C). Moreover, the liver ATGL protein levels in the mice fed on HFD tended to decline after 12 weeks and remarkably decreased after 16 weeks (Figure 1D), but there was no change in ATGL mRNA levels, as measured by quantitative real-time polymerase chain reaction (qRT-PCR) analysis (Figure 1E). Next, we analyzed two human liver tissue expression profiling datasets (GSE89632 and GSE48452) and found that liver ATGL mRNA levels were not significantly changed in the patients with hepatic steatosis (Figure 1F). In a hepatic steatosis cell model induced by 50 μM oleic acid (OA) treatment, ATGL protein level was noticeably downregulated, while ATGL mRNA level was not significantly changed after 15 days (Figure 1G and H). These results indicate the decline of ATGL protein levels in hepatic steatosis, which may be primarily attributed to the post-transcriptional regulation.

### ATGL is degraded through the proteasome pathway in hepatocytes

**Figure 2:**
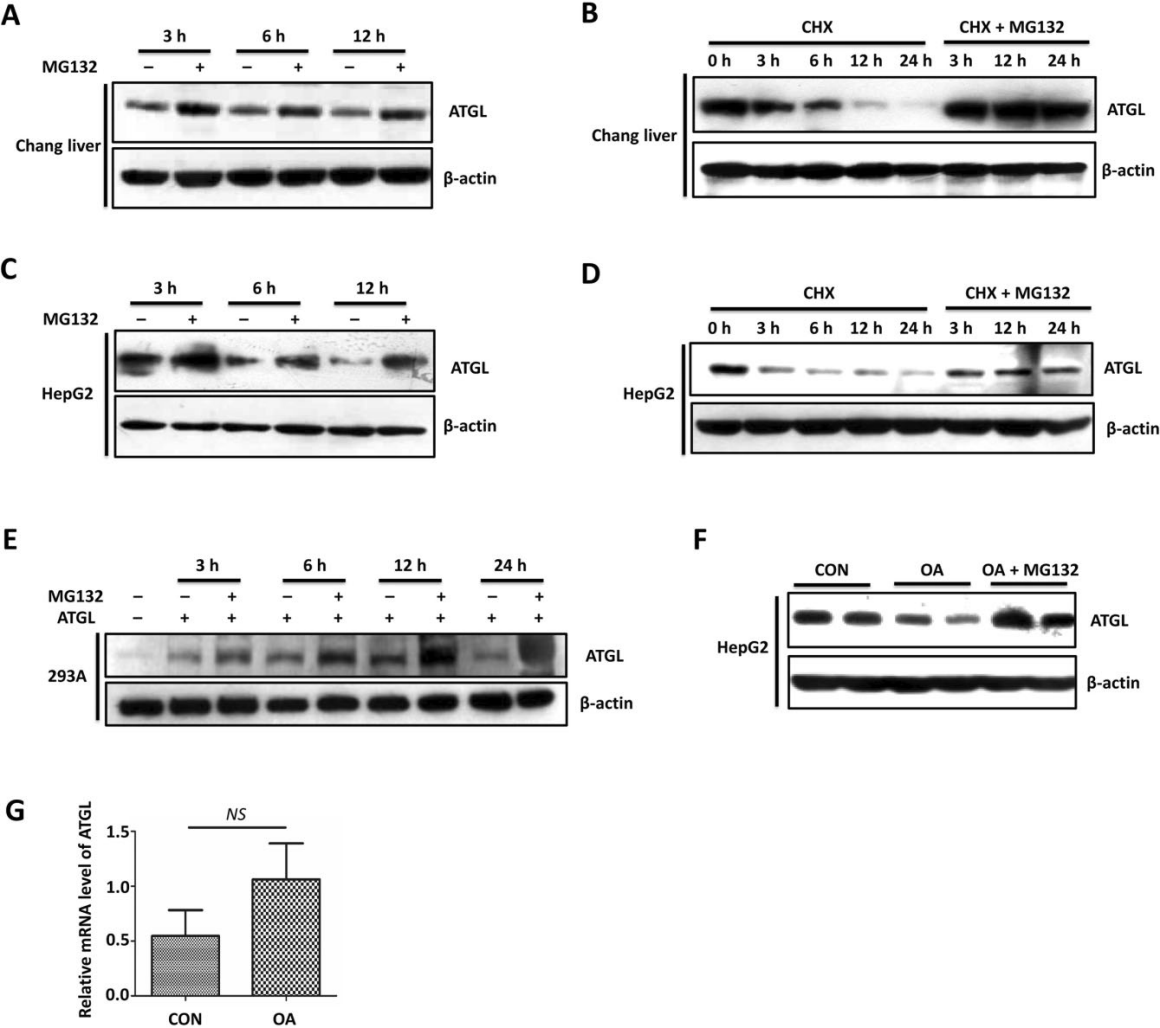
ATGL is degraded through the proteasome pathway in hepatic steatosis. (**A**–**D**) Chang liver (**A** and **B**) and HepG2 (**C** and **D**) cells were treated with 2 μg/ml CHX, 10 μM MG132, or a combination of CHX and MG132 for the indicated periods. (**E**) HEK293A cells were transfected with ATGL plasmids, followed by MG132 treatment for the indicated periods. (**F** and **G**) HepG2 cells were treated with 50 μM OA for 12 days, followed by MG132 treatment for 24 h. ATGL protein and mRNA levels were examined by western blotting and qRT-PCR, respectively. β-actin was used as a loading control. Data are representative of at least three independent experiments. Mean ± SEM; NS, no significance.

To evaluate whether the proteasome and/or lysosome pathways are involved in the downregulation of ATGL protein levels in hepatic steatosis, we treated cultured hepatocytes with the proteasome-specific inhibitor MG132 and the lysosomal inhibitor ammonium chloride (NH_4_Cl), respectively. While NH_4_Cl treatment did not change ATGL protein levels in Chang liver cells and HepG2 cells (Supplementary Figure S1), MG132 treatment led to higher ATGL protein levels in Chang liver, HepG2, and HEK293A cells (Figure 2A, C, and E), suggesting the involvement of the proteasome pathway in ATGL degradation. Then, we treated Chang liver cells and HepG2 cells with cycloheximide (CHX), which inhibits protein synthesis in eukaryotes, alone or in combination with MG132. As shown in Figure 2B and D, ATGL levels gradually decreased over time under CHX treatment but maintained invariably in the presence of MG132, confirming that ATGL is degraded through the proteasome pathway in hepatocytes. In addition, the ATGL protein levels, which were reduced in HepG2 cells treated with OA for 12 days without affecting the transcriptional level, could be restored by MG132 treatment (Figure 2F and G), suggesting that ATGL is also degraded through the proteasome pathway in hepatic steatosis.

### BTRC protein level is negatively correlated with ATGL protein level in hepatic steatosis

**Figure 3:**
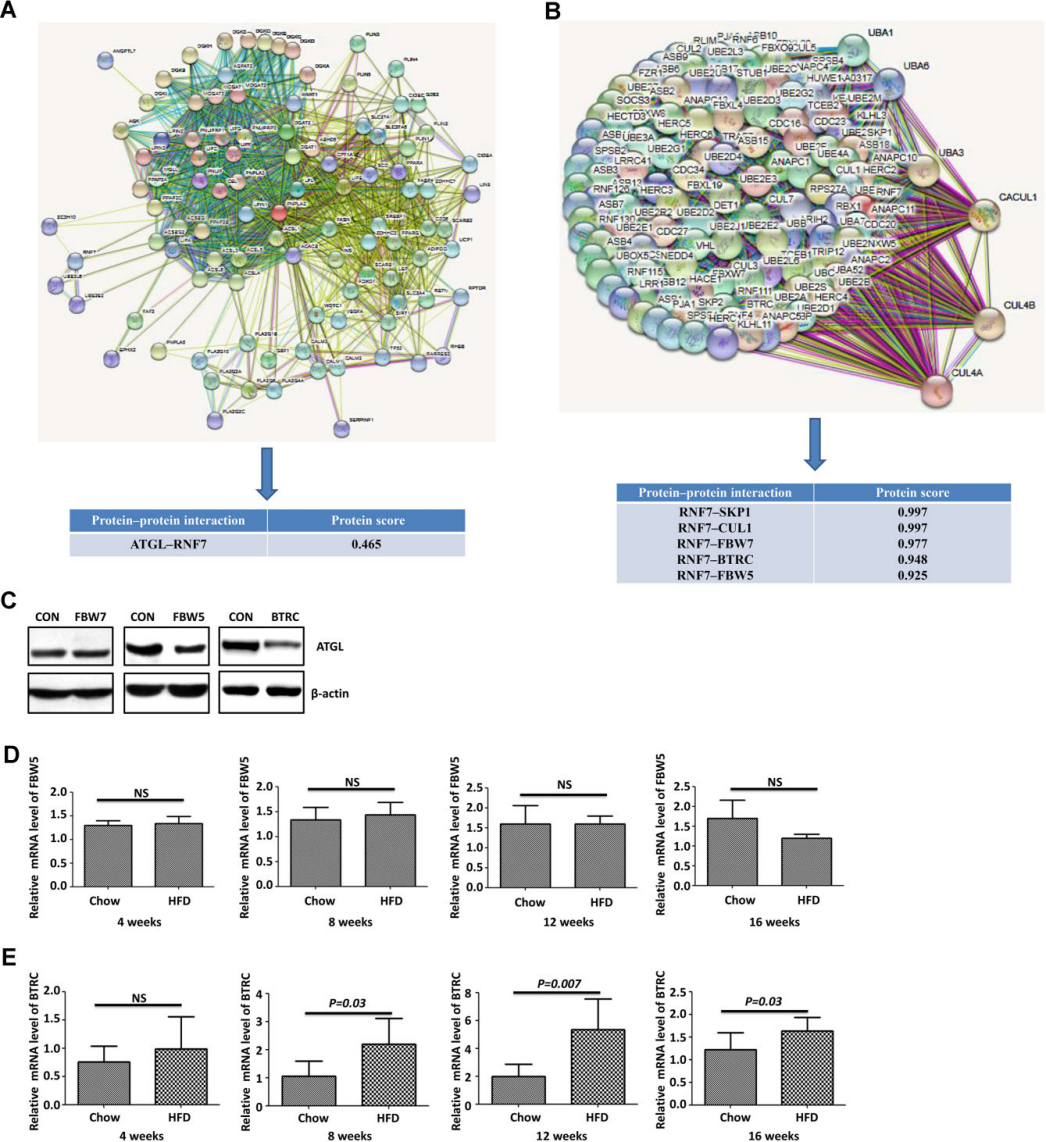
BTRC downregulates ATGL protein level. (**A** and **B**) Bioinformatics with the STRING database (https://string-db.org/) predicted interactions between RNF7 and ATGL, SKP1, CUL1, FBW7, BTRC, and FBW5, respectively. (**C**) HepG2 cells were transfected with FBW7, FBW5, and BTRC plasmids, respectively. After 24 h, ATGL protein levels were examined by western blotting. (**D** and **E**) FBW5 (**D**) and BTRC (**E**) mRNA levels in liver tissues of the mice fed on a chow diet or HFD for 4, 8, 12, and 16 weeks (*n* = 6 mice/group) were examined by qRT-PCR. Mean ± SEM; NS, no significance.

**Figure 4:**
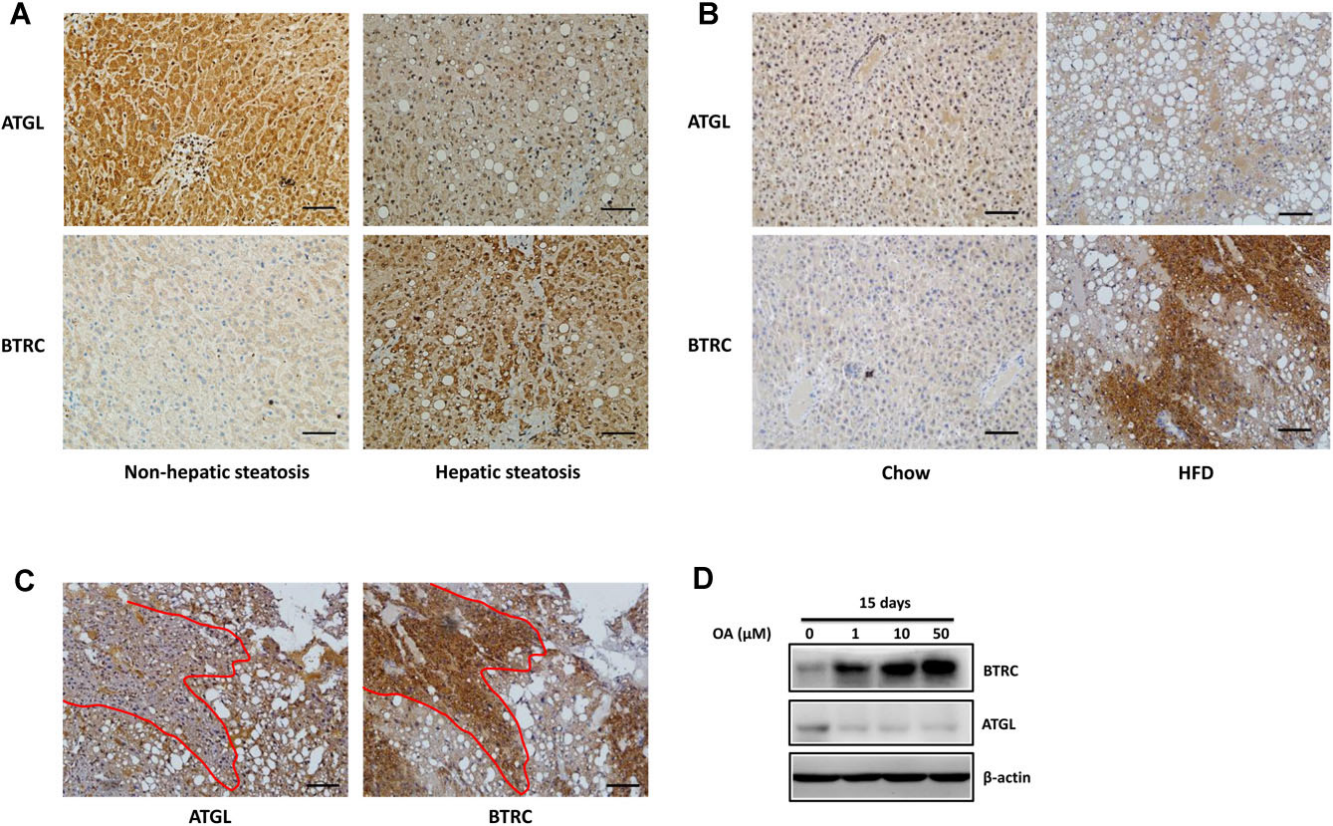
BTRC protein level is negatively correlated with ATGL protein level in hepatic steatosis. (**A**–**C**) IHC analysis for ATGL and BTRC in paracancerous liver tissues of hepatic carcinoma patients (**A**, *n =* 11 samples/group) and liver tissues of the mice fed on a chow diet or HFD for 16 weeks (**B** and **C**, *n =* 6 mice/group). (**C**) Co-staining of ATGL and BTRC in HFD-fed mouse liver. (**D**) HepG2 cells were treated with various concentrations of OA for 15 days. ATGL and BTRC protein levels were examined by western blotting. Scale bar, 100 μm (**A** and **B**) and 50 μm (**C**).

To identify the E3 ligase mediating ATGL proteasomal degradation, we first employed bioinformatics to predict proteins that may bind to ATGL. The STRING database (https://string-db.org/) indicated an interaction between ATGL and RNF7 (predicted interaction score 0.465, Figure 3A). RNF7 combines with several F-box proteins to form the S-phase kinase-associated protein 1 (SKP1)–Cullin 1 (CUL1)–F-box protein (SCF) Ub ligase complexes (Sun and Li, 2013; Zhou et al., 2013), which was also indicated by the STRING database (Figure 3B). Then, we examined ATGL protein levels in HepG2 cells overexpressing the F-box protein FBW7, FBW5, or BTRC, respectively. As shown in Figure 3C, overexpression of FBW5 and BTRC downregulated the ATGL protein level, while overexpression of FBW7 had no impact on ATGL. We further examined FBW5 and BTRC mRNA levels in mouse liver tissues and found that BTRC, but not FBW5, was upregulated in hepatic steatosis induced by HFD feeding (Figure 3D and E). Consistently, BTRC protein levels were increased in both human and mouse liver tissues with hepatic steatosis, where ATGL protein levels were decreased (Figure 4A and B). In particular, negatively correlated ATGL and BTRC staining could be observed in the mouse liver tissue with hepatic steatosis (Figure 4C). Furthermore, in HepG2 cells treated with various concentrations of OA for 15 days, BTRC protein levels were elevated, while ATGL protein levels were reduced (Figure 4D). Collectively, these results suggest that BTRC may be the E3 ligase that mediates the proteasomal degradation of ATGL in hepatic steatosis.

### BTRC acts as an E3 ligase of ATGL

**Figure 5:**
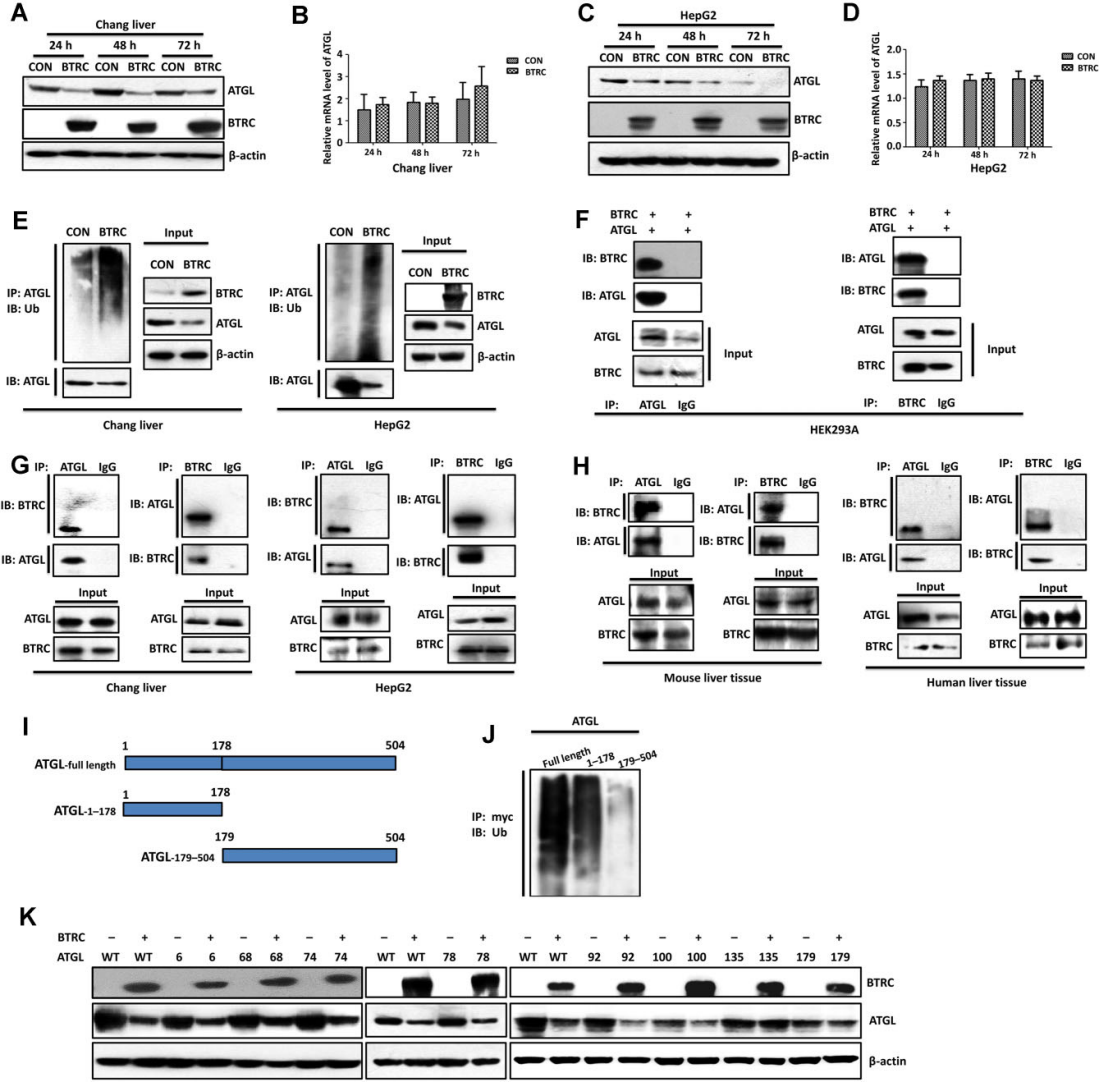
BTRC acts as an E3 ligase of ATGL. (**A**–**D**) Chang liver cells (**A** and **B**) and HepG2 cells (**C** and **D**) were transfected with BTRC plasmids. After 24, 48, and 72 h, ATGL protein and mRNA levels were examined by western blotting and qRT-PCR, respectively. (**E**) Chang liver cells and HepG2 cells were transfected with BTRC plasmids. After 48 h, cell lysates were prepared for IP with anti-ATGL antibody. The cell lysates and immunoprecipitates were analyzed by immunoblotting with anti-Ub and anti-ATGL antibodies. (**F**–**H**) Co-IP for BTRC and ATGL binding in lysates from HEK293A cells co-expressing BTRC and ATGL (**F**), Chang liver cells and HepG2 cells (**G**), and mouse and human liver tissues (**H**). IgG was used as the negative control. The co-IP assays were repeated independently three times and showed consistent results. (**I**) A graphical representation of full-length, 1–178 fragment, and 179–504 fragment of ATGL. (**J**) HepG2 cells were transfected with full-length, 1–178 fragment, and 179–504 fragment of ATGL. After 48 h, cell lysates were prepared for IP with anti-myc antibody. The cell lysates and immunoprecipitates were analyzed by immunoblotting with anti-Ub and anti-ATGL antibodies. (**K**) HepG2 cells were transfected with the indicated plasmids. ATGL protein levels were examined by western blotting. WT indicates wild-type ATGL; the number indicates the ATGL with this lysine residue mutated to arginine.

To verify that BTRC acts as an E3 ligase for ATGL proteasomal degradation, we first examined the effects of BTRC overexpression on ATGL expression and ubiquitination in Chang liver cells and HepG2 cells (Figure 5A–E). In both cell lines, BTRC overexpression resulted in markedly reduced ATGL protein levels but did not affect ATGL mRNA levels (Figure 5A–D), suggesting that BTRC regulated ATGL expression at the post-transcriptional rather than transcriptional level. BTRC overexpression also led to dramatically enhanced ATGL ubiquitination (Figure 5E). Next, we performed the co-immunoprecipitation (co-IP) assay with HEK293A cells overexpressing ATGL and BTRC (Figure 5F) or hepatocytes and liver tissues containing endogenous ATGL and BTRC (Figure 5G and H). The results demonstrate that BTRC directly binds to ATGL and acts as an E3 Ub ligase of ATGL.

### BTRC promotes ATGL ubiquitination and degradation predominantly at the lysine 135 residue

Subsequently, we sought to identify the lysine residues involved in BTRC-mediated ubiquitination and degradation of ATGL. We first examined the polyubiquitination levels of HepG2 cells transfected with myc-tagged vectors containing full-length (1–504 amino acids), 1–178 fragment (patatin-like, 1–178 amino acids), or 179–504 fragment (179–504 amino acids) of ATGL (Figure 5I) and found that the patatin-like fragment was critical for ATGL ubiquitination (Figure 5J). Then, we mutated lysine residues in (and adjacent to) the patatin-like fragment (Lys6, Lys68, Lys74, Lys78, Lys92, Lys100, Lys135, and Lys179, respectively) to arginine and detected ATGL protein levels in HepG2 cells overexpressing wild-type or mutant ATGL. As shown in Figure 5K, ATGL degradation by BTRC was only eliminated when the 135 residue was mutated.

### BTRC decreases lipolysis and increases lipid accumulation in the OA-induced hepatic steatosis cell model

**Figure 6:**
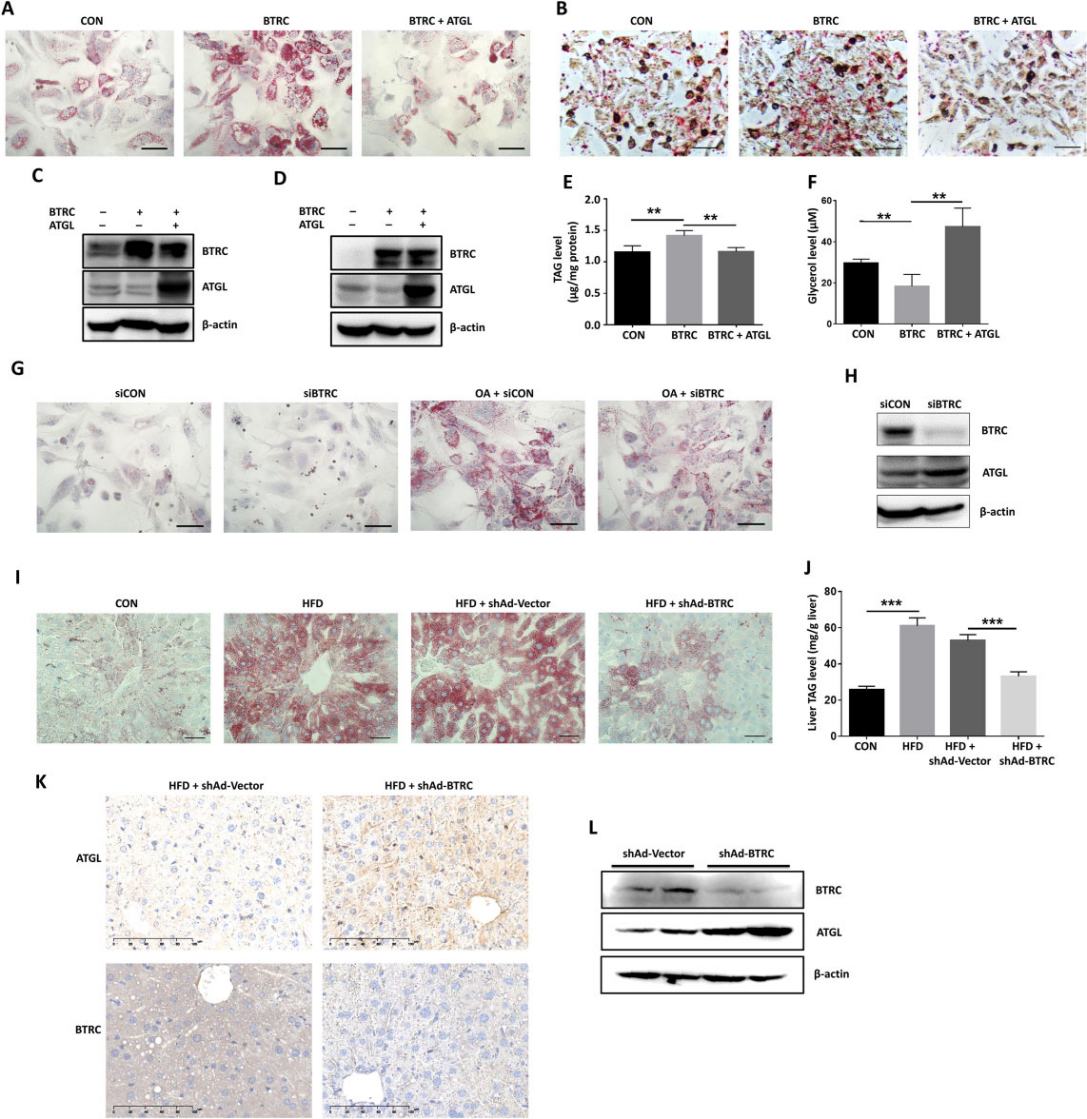
BTRC controls lipid accumulation and lipolysis by targeting ATGL for proteasomal degradation. (**A**–**F**) Primary hepatocytes (**A** and **C**) and HepG2 cells (**B** and **D**–**F**) were treated with 100 μM OA for 48 h to induce hepatic steatosis and then transfected with the indicated plasmids. (**A** and **B**) LD accumulation was assessed by Oil Red O staining. (**C** and **D**) ATGL and BTRC protein levels were examined by western blotting. (**E** and **F**) Cellular TAG (**E**) and glycerol (**F**) concentrations were determined by commercial kits. (**G** and **H**) OA-treated primary hepatocytes were transfected with siRNA for BTRC knockdown (siBTRC) or control siRNA (siCON). After 48 h, LD accumulation was assessed by Oil Red O staining (**G**), and ATGL and BTRC protein levels were detected by western blotting (**H**). (**I**–**L**) Mice were fed on HFD for 6 weeks to induce hepatic steatosis and then injected with shAd-BTRC or shAd-Vector via tail vein (*n* = 5 mice/group). After 3 weeks, hepatic LD accumulation (**I**), liver TAG level (**J**), and ATGL and BTRC protein levels (**K** and **L**) were examined. Scale bar, 50 μm (**A, B, G, I**) and 100 μm (**K**). Mean ± SEM; **P < 0.01, ***P < 0.001.

Primary hepatocytes or HepG2 cells were treated with OA for 48 h to induce hepatic steatosis and then transfected with BTRC plasmids, alone or together with ATGL plasmids, to evaluate the effects of BTRC overexpression on lipid accumulation and lipolysis (Figure 6A–F). After 48 h, the cells were subjected to Oil Red O staining, and the results revealed that overexpression of BTRC further enhanced cellular LD accumulation, which was reversed by co-expression of ATGL (Figure 6A–D). Similarly, BTRC-induced higher TAG and lower glycerol levels in HepG2 cells were also reversed by ATGL (Figure 6E and F). In contrast, knocking down BTRC by small interfering RNA (siRNA) reduced LD accumulation in the OA-induced hepatic steatosis cell model (Figure 6G and H; Supplementary Figure S2). These results indicate that BTRC decreases lipolysis and increases lipid accumulation via targeting ATGL for degradation.

### BTRC knockdown ameliorates hepatic steatosis in HFD-fed mice

Mice were fed on a chow diet or HFD for 6 weeks and then received a single injection with adenoviruses targeting BTRC (shAd-BTRC) or control adenoviruses (shAd-Vector). After another 3 weeks, we found that LD accumulation and TAG levels in the liver tissues of shAd-BTRC-injected HFD-fed mice were significantly lower than those in shAd-Vector-injected or untreated HFD-fed mice (Figure 6I and J). Besides, BTRC protein levels were significantly decreased, whereas ATGL protein levels were increased in the shAd-BTRC-injected mouse livers (Figure 6K and L). However, the plasma FFA and TAG levels were not significantly changed in shAd-BTRC-injected mice (Supplementary Figure S3). Similar results were obtained from another experiment where the mice received the adenovirus injection and were fed on HFD for 4 weeks (Supplementary Figure S4). Collectively, these results demonstrate that knockdown of BTRC increases hepatic ATGL protein levels and inhibits liver LD and TAG accumulation in the HFD-induced hepatic steatosis mouse model.

## Discussion

NAFLD patients with insulin resistance have higher liver steatosis grades and lower ATGL expression levels (Kato et al., 2008). Reid et al. (2008) reported that adenovirus-mediated hepatic overexpression of ATGL reduced liver TAG levels by 40%–60% in both ob/ob mice and HFD-induced obese mice. In agreement with previous findings, Ong et al. (2011) reported that the TAG hydrolase activity was reduced in the mice with adenovirus-mediated hepatic ATGL knockdown, resulting in a >2-fold increase in TAG content in the livers compared to controls. Turpin et al. (2011) observed that administering ATGL adenovirus not only reduced hepatic TAG, diacylglycerol, and ceramide contents but also enhanced insulin signal transduction in obese mice. Collectively, these findings suggest that treating hepatic steatosis requires the upregulation of ATGL expression, which is negatively correlated with hepatic diacylglycerol content. Consistently, we showed significantly downregulated ATGL protein levels in cholangioma and hepatic carcinoma patients with hepatic steatosis, HFD-induced hepatic steatosis mouse model, and OA-induced hepatic steatosis cell model (Figure 1). Protein expression level is generally regulated at the transcriptional or post-transcriptional level, and the latter involves the regulation of cellular protein turnover through the proteasome and lysosome pathways (Wilson and Cerione, 2000). Our previous work indicated that ATGL degradation in adipocytes was promoted by pigment epithelium-derived factor (PEDF) through the Ub-dependent proteasome pathway (Dai et al., 2013). Here, the results indicate that hepatic ATGL degradation is also regulated by the proteasome pathway involving the E3 ligase.

The SCF Ub ligase complex is composed of SKP1, CUL1, a variable member of the F-box protein family, and the RBX/ROC RING component (RBX1/ROC1 or RBX2/ROC2/SAG/RNF7) (Lau et al., 2012; Sun and Li, 2013). SCF complexes are the most prominent family of E3 ligases that ubiquitinate various regulatory proteins for 26S proteasomal degradation to regulate multiple biological processes, such as apoptosis, development, lipid metabolism, etc. (Lau et al., 2012; Zhou et al., 2013). One of the F-box proteins, BTRC, also called β-TrCP, has been demonstrated to regulate cellular processes by recognizing numerous essential molecules, including IκBα and β-catenin, for degradation and play a significant role in cancer development and inflammation (Frescas and Pagano, 2008; Lau et al., 2012; Bi et al., 2021). Another F-box protein, FBW7, was reported to promote the degradation of the SREBP family of transcription factors and regulate lipogenesis (Sundqvist et al., 2005; Onoyama et al., 2011). Here, we identified that BTRC, but not FBW7, serves as an ATGL E3 ligase in hepatocytes.

Ghosh et al. (2016) reported that the E3 Ub ligase constitutive photomorphogenic 1 (COP1), also known as RFWD2, could promote hepatic TAG accumulation by targeting ATGL proteasomal degradation predominantly at the lysine 100 residue, and adenovirus-mediated depletion of COP1 reduced hepatic lipid accumulation and improved liver function in HFD-fed mice (Ghosh et al., 2016). PEDF also diminishes ATGL protein stability by promoting its proteasomal degradation in a COP1-dependent manner (Niyogi et al., 2019). However, we showed that BTRC promotes ATGL degradation predominantly at the lysine 135 residue (Figure 5K), suggesting that BTRC is a new E3 ligase for ATGL. Moreover, our results demonstrated that BTRC was significantly upregulated in both human and mouse liver tissues with hepatic steatosis, suggesting a critical role of BTRC in the development of NAFLD.

Apart from ATGL degradation, the continuously elevated BTRC levels in hepatic steatosis also promote liver inflammation. As an E3 ligase for IκBα, BTRC can induce the nuclear translocation of NF-κB, enabling the expression of target genes that encode inflammatory mediators (Rahman and McFadden, 2011), and eventually lead to NASH. Moreover, previous studies demonstrated that NF-E2 p45-related factor 2 (Nrf2) is downregulated during the development of NASH, whereas in pre-clinical studies, the upregulation of Nrf2 inhibits NASH (Bathish et al., 2022). BTRC could inhibit the activity of Nrf2 through GSK-3β (Salazar et al., 2006), suggesting a role of BTRC in the pathophysiology of NASH, and thus it can be a potential therapeutic target for treating NAFLD. PROteolysis TArgeting Chimeras (PROTAC) technology is an emerging protein degradation technology that has incomparable potential advantages over the use of small-molecule inhibitors, such as targeting non-patent targets and overcoming drug resistance. The technology has been extensively studied in the treatment of various diseases such as cancers, viral infections, and neurodegenerative diseases (Guedeney et al., 2023). In the further studies, PROTAC technology may offer an effective approach for treating NAFLD by targeting BTRC degradation.

## Materials and methods

### Antibodies and reagents

Rabbit anti-ATGL, mouse anti-Ub, and rabbit anti-BTRC antibodies were from Cell Signaling Technology; DAPI and β-actin antibody were from Thermo Scientific; MG-132 was from Merck (catalog number 47490); and the lysosomal inhibitor NH_4_Cl was from Sigma-Aldrich (catalog number 254134).

### Cell culture and transfection

Human hepatoma cell line HepG2 and human embryonic kidney cell line HEK293A obtained from ATCC and human liver cell line Chang liver obtained from the Cell Bank of Chinese Academy of Sciences (Shanghai) were cultured in high-glucose Dulbecco's modified Eagle's medium (DMEM) supplemented with 10% fetal bovine serum (FBS) and 1% penicillin/streptomycin. Primary hepatocytes were isolated by perfusing mouse liver with 0.4 mg/ml collagenase IV (Sigma, catalog number C5138) and cultured in Willian's E medium with hepatocyte maintenance culture supplement containing 10% FBS and 1% penicillin/streptomycin. Transient transfections with plasmids and siRNAs were performed using Lipofectamine 2000 reagent (Thermo Fisher Scientific, catalog number 11668027) according to the manufacturer's instruction.

### Plasmids

Human BTRC, FBW7, FBW5, and ATGL plasmids were constructed using Ruyilian Kit (SiDanSai Biotechnology) according to the manufacturer's instruction. BTRC siRNA was purchased from RiboBio Company. ATGL clone mutagenesis was performed using In-Fusion HD Cloning Kit (Clontech, catalog number 639650). Details are described in Supplementary material.

### Animal experiments

All animal experiments were reviewed and approved by the Animal Ethics Committee of Sun Yat-sen University. The use and handling of animals were performed strictly in accordance with the Guidelines for the Use of Laboratory Animals by Sun Yat-sen University.

Male C57BL/6J mice (7–8 weeks old) were obtained from Vital River, housed under specific pathogen-free conditions with a 12-h light/dark cycle, a controlled humidity (40%–70%), and a stable temperature (22°C ± 3°C), and fed a chow diet or HFD (Research Diets, D12492). The adenoviruses for BTRC knockdown and control adenoviruses were obtained from OBiO. To verify the interfering effect, the viruses were used to infect Hepa1-6 cells at a multiplicity of infection of 10. For animal experiments, viruses were diluted in phosphate-buffered saline and administered into mice via tail vein (1 × 10^9^ plaque-forming units per mouse).

Male naturally obese C57BL/6J mice (6 months old) obtained from Vital River were all fed on a regular diet. The most obese mice were selected for the hepatic steatosis group (38.16 ± 0.12 g), while the mice with average body weight were selected for the control group (30.44 ± 0.44 g).

### Measurement of TAG, FFA, and glycerol levels

Lipids were extracted from liver tissues or cultured hepatocytes using chloroform/methanol (2:1, *v*/*v*) as previously described (Kim et al., 2010). The amounts of TAG, glycerol, and FFA were measured using EnzyChrom^TM^ Triglyceride Assay Kit, Glycerol Assay Kit, and Free Fatty Acid Assay Kit, respectively (BioAssay Systems).

### Histological analysis of liver tissues

All human samples were collected with patients’ consent, and the experiments were approved by the Ethics Committee of Sun Yat-sen University. The surgical liver specimens of cholangioma patients (from The First Affiliated Hospital of Sun Yat-sen University) and hepatocellular carcinoma patients (from The Second Affiliated Hospital of Sun Yat-sen University) with hepatic steatosis or non-hepatic steatosis, as well as mouse liver samples, were fixed with 4% (*w*/*v*) paraformaldehyde overnight. Sections (5-μm thick) were prepared from the paraffin-embedded tissues and stained for ATGL and BTRC, respectively. Detection was performed using the ABC HRP Kit (Vector Laboratories) with 3,3′-diaminobenzidine, followed by counterstaining with hematoxylin or methyl green. Images were obtained using a light microscope (Nikon Corporation).

### qRT-PCR

Total RNA was isolated using TRIzol (Invitrogen) and then reverse-transcribed for qRT-PCR analysis, with primers as follows: BTRC, 5′-GGAGAAGACTTTGACCAGCG-3′ and 5′-CTTTGGAATTCGAGTCGAGC-3′; FB5, 5′-GGAGAAGACTTTGACCAGCG-3′ and 5′-CTTTGGAATTCGAGTCGAGC-3′; and FB7, 5′-GGAGAAGACTTTGACCAGCG-3′ and 5′-CTTTGGAATTCGAGTCGAGC-3′. Amplification reactions were performed with an initial denaturation step at 94°C for 2 min, followed by 40 cycles of denaturation at 94°C for 5 min, annealing at 55°C for 30 sec, and extension at 72°C for 2 min.

### Co-IP

For IP, 1000 μg of tissue homogenate or cell lysates were incubated with 8 μg of anti-ATGL or anti-BTRC antibody overnight at 4°C, followed by the addition of 40 μl Protein A/G Sepharose beads (Calbiochem) and incubation for 4 h at 4°C. Immunoprecipitates were washed four times with lysis buffer, eluted with loading buffer, and analyzed by western blotting.

### OA-induced hepatic steatosis cell model

HepG2 cells were treated with the indicated concentration of OA solution (Sigma-Aldrich) and cultured in DMEM (HyClone; GE Healthcare Life Sciences) with 0.2% FBS. After different periods, the medium was removed, and the cells were harvested for western blotting or Oil Red O staining.

### Oil red O staining

Oil Red O staining was performed as described before (Xu et al., 2014). Photographs were taken with a camera under optimal illumination.

### Statistical analysis

Student's unpaired two-tailed *t*-test was used to evaluate the statistical significance between two groups; for more than two groups, analysis of variation was used, followed by Fisher's least significant difference test. Statistical significance was predefined as *P* < 0.05.

## Supplementary Material

mjad064_Supplemental_File

## Notes

**Author contributions:** G.G. and W.Q. designed and performed experiments, analyzed data, and wrote the manuscript. X.Y. and Y.X. designed and performed experiments and wrote the manuscript. Z.F., C.L., Y.Z., and Y.L. performed experiments and assessed the outcome. H.H. provided human samples and designed experiments. Z.D. designed experiments. T.Z. and J.L. designed experiments, analyzed data, and wrote the manuscript. All authors reviewed and commented on the manuscript.
