# Supplementary material for: The critical role of BTRC in hepatic steatosis as an ATGL E3 ligase
Source: J Mol Cell Biol. 2023 Oct 23;15(10):mjad064. doi: 10.1093/jmcb/mjad064 (PMC10993717; doi:10.1093/jmcb/mjad064)
Supplement: mjad064_Supplemental_File [file mjad064_supplemental_file.pdf]

Supplementary material

The critical role of BTRC in hepatic steatosis as an ATGL E3 ligase

Weiwei Qi[1,†], Zhenzhen Fang[1,†], Chuanghua Luo[1,†], Honghai Hong[1,2,†], Yanlan Long[1], Zhiyu Dai[3], Junxi Liu[1], Yongcheng Zeng[1], Ti Zhou[1], Yong Xia[2,*], Xia Yang[1,4,*], and Guoquan Gao[1,5,6,*]

[1] Department of Biochemistry, Zhongshan School of Medicine, Sun Yat-sen University, Guangzhou 510080, China

[2] Department of Clinical Laboratory, The Third Affiliated Hospital of Guangzhou Medical University, Guangzhou 510006, China

[3] Department of Internal Medicine, University of Arizona College of Medicine, Phoenix, AZ 85004, USA

[4] Guangdong Engineering & Technology Research Center for Gene Manipulation and Biomacromolecular Products, Sun Yat-sen University, Guangzhou 510080, China

[5] Guangdong Province Key Laboratory of Brain Function and Disease, Zhongshan School of Medicine, Sun Yat-sen University, Guangzhou 510080, China

[6] Key Laboratory of Tropical Disease Control (Sun Yat-sen University), Ministry of Education, Guangzhou 510080, China

[†] These authors contributed equally to this work.

* Correspondence to: Guoquan Gao, Tel/Fax: 86-20-87332128, E-mail: gaogq@mail.sysu.edu.cn; Xia Yang, E-mail: yangxia@mail.sysu.edu.cn; Yong Xia, E-mail: 377695944@qq.com

Plasmids and vectors

Human BTRC, FBW7, FBW5 and ATGL clones were constructed using Ruyilian Kit from SiDanSai Biotechnology (Sidansai, Shanghai, China) according to the manufacturer’s instruction. In briefly, human BTRC, FBW7, FBW5 and ATGL were cloned from cDNA using specific primiers (Supplementary Table S1), and then fused to the vector provided by Ruyilian Kit. SiRNA for BTRC was purchased from Ribobio Company (Guangzhou, China). The sequence of the BTRC siRNA was 5'-GACTACAGTTTGATGAATT-3' (for human) and 5'-GCGACATAGTTTACAGAGA-3' (for mouse). Cells were transfected with the BTRC siRNA using Lipofectamine 2000 (Invitrogen, Carlsbad, CA, USA) according to the manufacturer's instructions. Mutagenesis of the ATGL clone was done using In-Fusion technology from Clontech Company (Catalog 639650, Clontech, Japan)

according to the manufacturer's instruction. In briefly, ATGL lysine site mutation fragment was cloned from ATGL plasmids using specific mutant primiers (Supplementary Table S2), and then connected with the linear vector cloning using corresponding primers (Supplementary Table S2).

Supplementary Tables

Supplementary Table S1. The primers for cloning human BTRC, FBW7, FBW5, and ATGL using Ruyilian Kit.

Genes	Primer
BTRC	Forward primer: 5'-gggggaagacggagtgatggacccggccgaggcggtg-3'<br>Reverse primer: 5'-gccggaagacaatgggttatctggagatgtaggtgtatgt-3'
FBW7	Forward primer: 5'-gggggaagacggagtgatgaatcaggaactgctctctgtg-3'<br>Reverse primer: 5'-gccggaagacaatgggtcacttcatgtccacatcaaagtccag-3'
FBW5	Forward primer: 5'-gggggaagacggagtgatggacgagggcggcacgcccc-3'<br>Reverse primer: 5'-gccggaagacaatgggttagcgcctctggctggcaagccag-3'
ATGL	Forward primer: 5'-gggggaagacggagtgatgtttccccgcgagaagacgtgg-3'<br>Reverse primer: 5'-gccggaagacaatgggttacagccccagggccccgatcacg-3'

Supplementary Table S2. The primers for ATGL lysine site mutation and corresponding primers for linear vector.

Site mutation	Primers for ATGL lysine site mutation		Corresponding primers for linear vector	
	Forward primer	Inverse primer	Forward primer	Inverse primer
The 6th Lys→Arg mutation	5'-gccacgtggaacatctc gttcgcggg-3'	5'-caaggaaggcacgggg gagggcaa-3'	5'-cccgtgccttccttgacc ctggaaggt-3'	5'-gatgttccacgtggcctc gcggggaaa-3'
The 68th Lys→Arg mutation	5'-gccttcattgaggtatcta aagaggccc-3'	5'-caaggaaggcacgggg gagggcaa-3'	5'-cccgtgccttccttgacc ctggaaggt-3'	5'-cttccgggcctcggcag atacctcaatgaa-3'
The 74th Lys→Arg mutation	5'-gccgaggcccggaagc ggttcctgggc-3'	5'-caaggaaggcacgggg gagggcaa-3'	5'-cccgtgccttccttgacc ctggaaggt-3'	5'-gcccaggaaccgggcc cggg cctctttaga-3'
The 78th Lys→Arg mutation	5'-gcccggttcctgggccc cctgcacccc-3'	5'-caaggaaggcacgggg gagggcaa-3'	5'-cccgtgccttccttgacc ctggaaggt-3'	5'-actgcggatgatggcta ccaggttgaagga-3'
The 100th Lys→Arg mutation	5'-gccatcatccgcagtttc ctgctgaag-3'	5'-caaggaaggcacgggg gagggcaa-3'	5'-cccgtgccttccttgacc ctggaaggt-3'	5'-actgcggatgatggcta ccaggttgaagga -3'
The 135th Lys→Arg mutation	5'-gccgtcctgcctgctgat agccatgag-3'	5'-caaggaaggcacgggg gagggcaa-3'	5'-cccgtgccttccttgacc ctggaaggt-3'	5'-agcaggcaggacggcc agcaggaaactgcg-3'
The 179th Lys→Arg mutation	5'-gccgacgagctcatcca ggccaatgtc-3'	5'-caaggaaggcacgggg gagggcaa-3'	5'-cccgtgccttccttgacc ctggaaggt-3'	5'-gatgagctcgtcggcgg agttgaagtggga-3'

Supplementary Figures

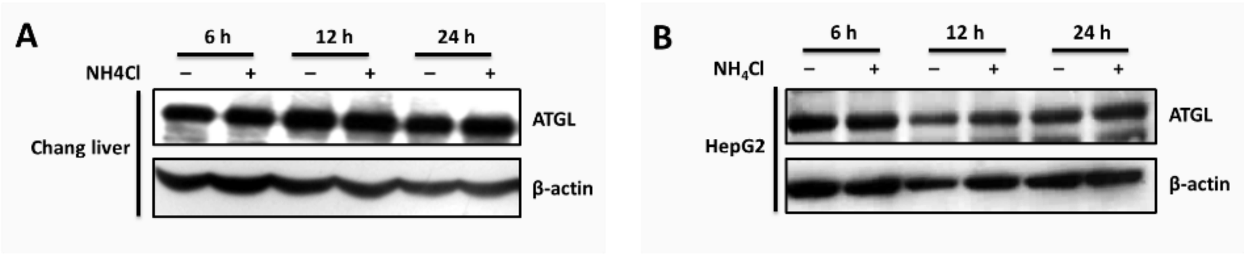

Supplementary Figure S1. The level of ATGL in Chang liver and HepG2 cells with the treatment of lysosomal inhibitor ammonium chloride.

ATGL was detected by immunoblotting in Chang liver cells (**A**) and HepG2 cells (**B**) treated with ammonium chloride (NH_4Cl, 10 mM) for indicated time. β-actin was used as a loading control. Data are representative of at least three independent experiments.

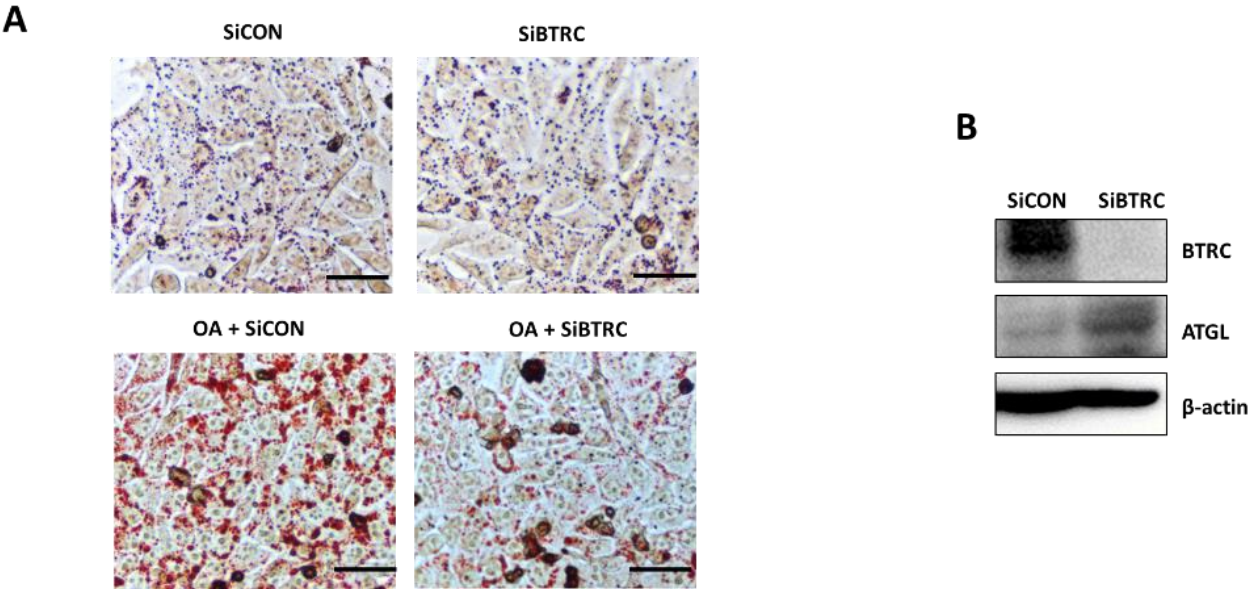

Supplementary Figure S2. After interfering with BTRC by siRNA, lipid droplet accumulation was reduced in oleic acid-induced HepG2 cells.

OA-induced HepG2 cells were interfered with BTRC by siRNA for 48 h, followed by Oil Red O staining (**A**) and ATGL and BTRC detection by western blot analysis (**B**). Scale bar, 50 µm.

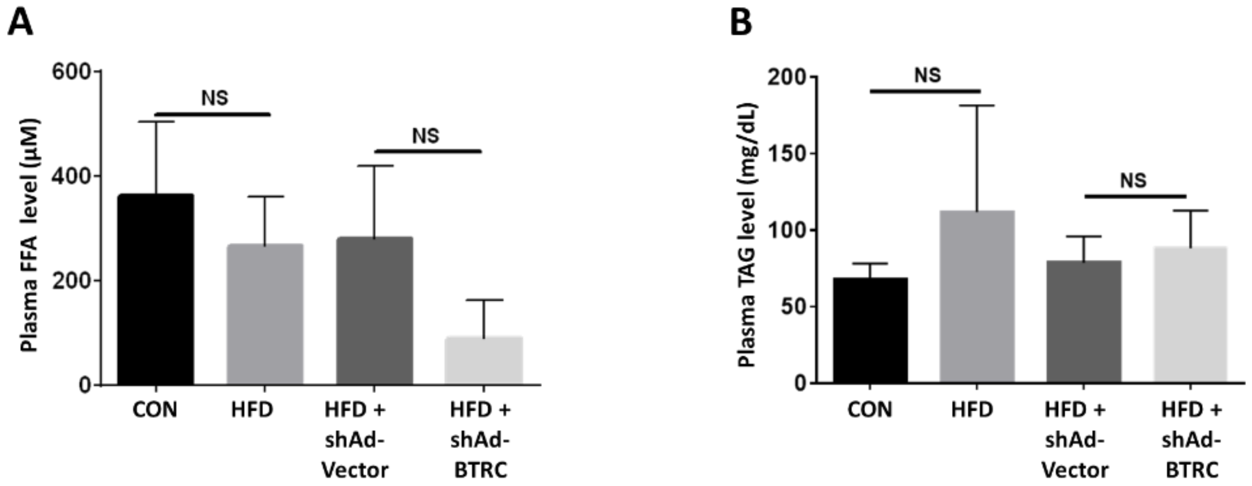

Supplementary Figure S3. FFA and TAG levels in the plasma of shAd-BTRC-injected animals. After six weeks of high fat feeding, the mice was injected with adenovirus specifically knocking down BTRC (shAd-BTRC) for three weeks. FFA level (**A**) and TAG level (**B**) in the plasma were detected by commercialized kits. Data are represented as mean ± SEM of three independent assays.

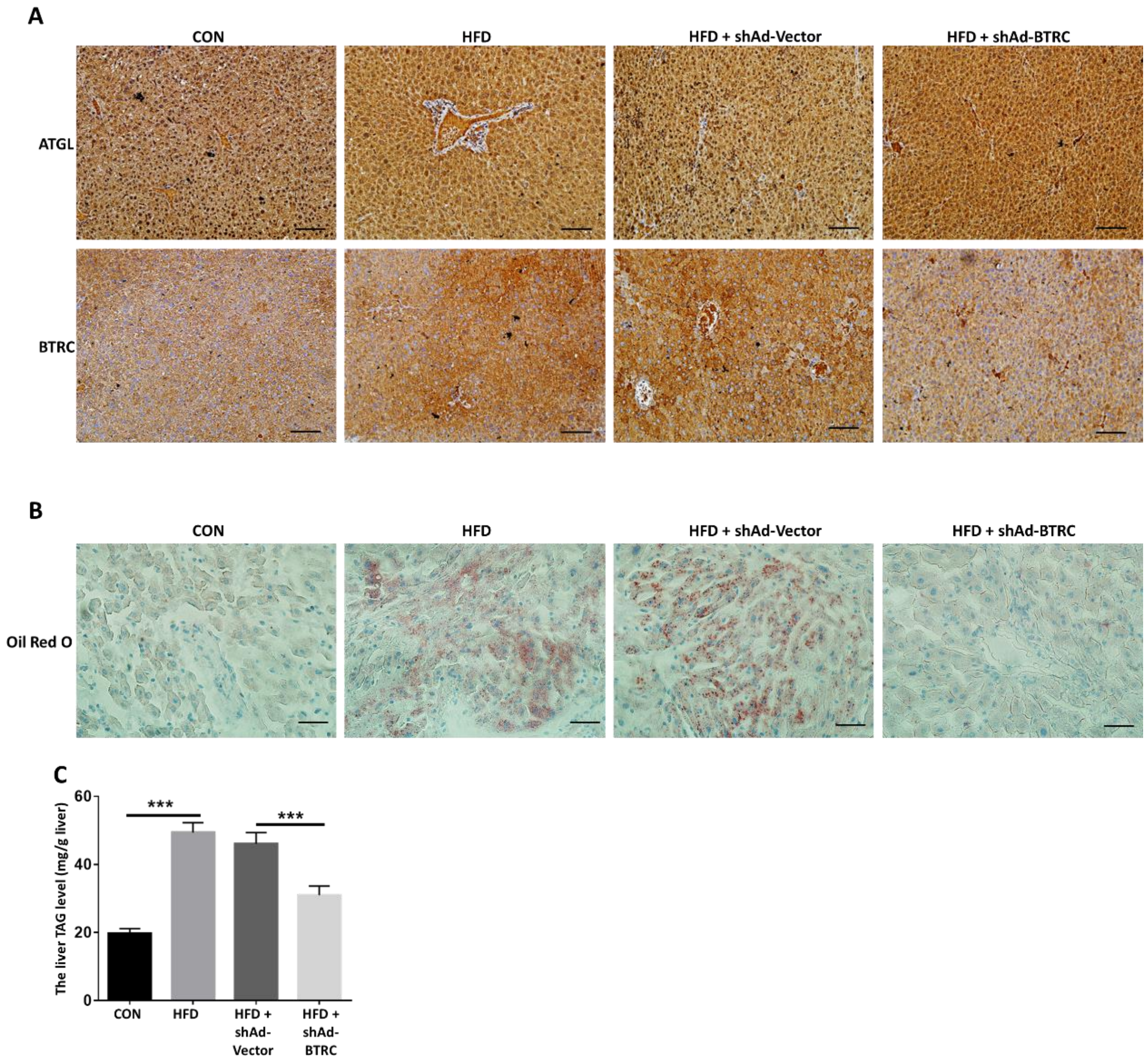

Supplementary Figure S4. BTRC knockdown ameliorates hepatic steatosis in high-fat diet mouse.

(**A** and **B**) The mice were injected with shAd-BTRC adenovirus and fed high-fat diet for four weeks simultaneously, followed by the assessment of ATGL and BTRC expression by immunohistologic staining (**A**) and lipid droplet accumulation analysis by Oil Red O staining (**B**). Scale bar, 100 μm (**A**) and 50 μm (**B**). (**C**) The TAG level in the liver was detected by commercialized kit. Data are represented as mean ± SEM of three independent assays. **$P < 0.01$; ***$P < 0.001$. $n = 5$ mice/group.
